# Prolonged sleep restriction induces changes in pathways involved in cholesterol metabolism and inflammatory responses

**DOI:** 10.1038/srep24828

**Published:** 2016-04-22

**Authors:** Vilma Aho, Hanna M. Ollila, Erkki Kronholm, Isabel Bondia-Pons, Pasi Soininen, Antti J. Kangas, Mika Hilvo, Ilkka Seppälä, Johannes Kettunen, Mervi Oikonen, Emma Raitoharju, Tuulia Hyötyläinen, Mika Kähönen, Jorma S.A. Viikari, Mikko Härmä, Mikael Sallinen, Vesa M. Olkkonen, Harri Alenius, Matti Jauhiainen, Tiina Paunio, Terho Lehtimäki, Veikko Salomaa, Matej Orešič, Olli T. Raitakari, Mika Ala-Korpela, Tarja Porkka-Heiskanen

**Affiliations:** 1 Department of Physiology, Faculty of Medicine, University of Helsinki, Finland; 2 Genomics and Biomarkers unit and Institute for Molecular Medicine FIMM, National Institute for Health and Welfare, Helsinki, Finland; 3 Department of Psychiatry, University of Helsinki and Helsinki University Hospital, Finland; 4 Stanford University Center for Sleep Sciences, Palo Alto, CA, USA; 5 Department of Chronic Disease Prevention, Population Studies Unit, National Institute for Health and Welfare, Turku, Finland; 6 VTT Technical Research Centre of Finland, Espoo, Finland; 7 Steno Diabetes Center A/S, Gentofte, Denmark; 8 Computational Medicine, Institute of Health Sciences, University of Oulu, Oulu, Finland; 9 NMR Metabolomics Laboratory, School of Pharmacy, University of Eastern Finland, Kuopio, Finland; 10 Department of Clinical Chemistry, Fimlab Laboratories, and University of Tampere, School of Medicine, Tampere, Finland; 11 Research Centre of Applied and Preventive Cardiovascular Medicine, University of Turku, Turku, Finland; 12 Department of Clinical Physiology, University of Tampere and Tampere University Hospital, Tampere, Finland; 13 Department of Medicine, University of Turku, and Division of Medicine, Turku University Hospital, Turku, Finland; 14 Brain and Work Research Centre, Finnish Institute of Occupational Health, Helsinki, Finland; 15 Agora Center, University of Jyväskylä, Jyväskylä, Finland; 16 Minerva Foundation Institute for Medical Research, Helsinki, Finland; 17 Institute of Biomedicine, Anatomy, University of Helsinki, Finland; 18 Unit of Excellence for Immunotoxicology, Finnish Institute of Occupational Health, Helsinki, Finland; 19 Department of Chronic Disease Prevention, National Institute for Health and Welfare, Helsinki, Finland; 20 Department of Clinical Physiology and Nuclear Medicine, Turku University Hospital, Turku, Finland; 21 Oulu University Hospital, Oulu, Finland; 22 Computational Medicine, School of Social and Community Medicine & Medical Research Council Integrative Epidemiology Unit, University of Bristol, Bristol, United Kingdom

## Abstract

Sleep loss and insufficient sleep are risk factors for cardiometabolic diseases, but data on how insufficient sleep contributes to these diseases are scarce. These questions were addressed using two approaches: an experimental, partial sleep restriction study (14 cases and 7 control subjects) with objective verification of sleep amount, and two independent epidemiological cohorts (altogether 2739 individuals) with questions of sleep insufficiency. In both approaches, blood transcriptome and serum metabolome were analysed. Sleep loss decreased the expression of genes encoding cholesterol transporters and increased expression in pathways involved in inflammatory responses in both paradigms. Metabolomic analyses revealed lower circulating large HDL in the population cohorts among subjects reporting insufficient sleep, while circulating LDL decreased in the experimental sleep restriction study. These findings suggest that prolonged sleep deprivation modifies inflammatory and cholesterol pathways at the level of gene expression and serum lipoproteins, inducing changes toward potentially higher risk for cardiometabolic diseases.

Short sleep duration, complaints of poor sleep quality and diagnosed sleep problems have in epidemiological studies been associated with metabolic disorders, which relate to low-grade chronic inflammation, including cardiovascular diseases, metabolic syndrome, obesity, and type 2 diabetes mellitus^1^,^2^,^3^,^4^. However, the functional pathways and molecules that mediate these effects are largely unknown.

Several previous studies have shown that experimental restriction of sleep to 4–5 h per night for 1–2 weeks activates immune responses^5^,^6^,^7^, down-regulates gene pathways for macromolecular biosynthesis and metabolic processes^8^, and modifies glucose metabolism by inducing insulin resistance^9^,^10^,^11^. However, the reported serum lipid levels, central factors in the pathogenesis of atherosclerosis, have shown only mild, inconsistent, or no effects^12^,^13^,^14^, leaving open the question what specific metabolic modulations induced by sleep restriction could explain the increased association of restricted sleep to atherosclerosis, which is characterised by slow build-up of lipid plaques in the walls of the arteries, promoted by inflammatory responses and decrease in macrophage reverse cholesterol transport (RCT)^15^.

The duration of sleep restriction in previous studies has ranged from one to five days, which in the development of chronic diseases is a short period. In trying to understand the role of restricted sleep as a predisposing factor for such diseases, the key questions are: how do the short-term modifications in metabolism and inflammation develop when the duration of the sleep restriction is prolonged, and are these modifications such that could explain the association between restricted sleep and increased risk for e.g. atherosclerosis, as evidenced by the epidemiological studies?

The assessment of circulating lipid profiles using nuclear magnetic resonance (NMR) spectroscopy goes beyond the typically measured total lipids, like total cholesterol and triglycerides, and allows detailed characterization of many lipoprotein features at the subclass level, including the size of the particles, which are important in lipid physiology and pathophysiology^16^,^17^. Analysis of circulating lipid molecules based on mass spectrometry (MS), which gives an extensive profile of individual lipid molecules but does not quantify lipoprotein-related measures, has previously been applied in circadian research^18^,^19^,^20^,^21^. Recently one short-term sleep deprivation study^22^ and one partial sleep restriction study^23^ have applied MS-based lipid analyses in serum, while one study used NMR to assess urine metabolites in short-term sleep deprivation^24^, but NMR-based lipoprotein subclass analyses have not been used to analyse the effects of sleep loss. The assessment of the relationship between sleep/sleep insufficiency and metabolomics profiles in large epidemiological cohorts has not, to our knowledge, been reported before.

The short-term effects of insufficient sleep were assessed in a highly controlled experiment where a group of volunteers restricted their sleep to 4 hours per night during 5 days (sleep restriction, SR, N = 14 cases and N = 7 controls). The results focusing on the immunological effects at the level of gene expression, cytokines, and CRP have been previously published^5^. The putative longer-term effects were assessed in real-life conditions using two independent epidemiological cohorts (DILGOM^25^, N = 518, and Young Finns Study, YFS^26^, N = 2221), where the insufficiency of sleep was evaluated based on self-reported sleep parameters (subjective sleep insufficiency, SSI). Genome-wide transcriptome and NMR-based metabolome were obtained from all three samples, and mass spectrometry-based lipidome from the SR study participants (see flow of the analyses depicted in Supplementary Fig. S1).

## Results

### Subjective sleep insufficiency in epidemiological cohorts

Partial sleep loss was induced experimentally as 4 h sleep/night for 5 nights for 14 healthy young males in the SR study (including also 7 control subjects; total N = 21; age (mean ± s.d.) 23.2 ± 2.2 y, Supplementary Table S1)^5^. To study sleep loss in real life conditions, subjective feeling of insufficient sleep was assessed using questionnaire information in two epidemiological samples. In the DILGOM subsample with information of subjective sleep sufficiency and omics data (N = 472, 46% men, age (mean ± s.d.) 51.9 ± 13.8 y, Supplementary Table S1)^25^, we used the question ”Do you, in your opinion, sleep enough?”. The answer options were dichotomised, combining subjects reporting to sleep enough “almost always” (N = 168) or “often” (N = 218) to a phenotype of ‘subjective sufficient sleep’ (noSSI, N = 386). Subjects reporting to “seldom or almost never” (N = 86) sleep enough were considered as having ‘subjective sleep insufficiency’ (SSI, N = 86, 18.2%).

In the Cardiovascular Risk in Young Finns Study (“Young Finns Study”, YFS; N = 2221, 55% men, age (mean ± s.d.) 37.7 ± 5.0 y, Supplementary Table S1) replication cohort^26^, sufficiency of sleep was assessed using two questions: one addressing self-reported sleep duration (“How many hours do you usually sleep per night?”) and another on self-reported sleep need (“How many hours of sleep do you need per day to feel well rested?”). Subjective sleep duration was subtracted from subjective sleep need, and individuals sleeping more than an hour over their sleep need (N = 37, 1.7%) were excluded from further analyses. Remaining subjects were grouped into three groups based on their level of SSI: no (or only mild) SSI (sleep need – sleep duration = −1…0…1 h; N = 1825, 82.2%), moderate SSI (1.5–2 h; N = 304, 13.7%), and heavy SSI (>2 h; N = 55, 2.5%). Despite the differences in the questions and age groups, the overall prevalence of SSI (mSSI or hSSI), 16.2%, in the YFS sample was quite similar to the 18.2% found in the DILGOM. In accordance with these results, the prevalence of “sleep debt”, using closely similar criterion as used in the YFS sample, has been earlier found to be 20% in 1004 French young adults^27^.

### Gene expression in lipid pathways

#### Pathway analysis of differentially expressed genes

Gene expression profiles were analysed from peripheral blood mononuclear cells (PBMC) in the SR study (N = 9 cases, N = 4 controls)^5^ and whole blood in the DILGOM cohort (N = 472) using microarrays. Lipid-related pathways were enriched among transcripts down-regulated after 5 nights of experimental SR^5^. In the epidemiological DILGOM sample, linear regression was used to correlate RNA expression with SSI, adjusting for age and gender. Transcripts from 725 genes (2% of the total 35,420 transcripts analysed) had lower expression among subjects with SSI (N = 86) compared to subjects with no sleep complaints (N = 386) (pointwise *P* < 0.05). Enrichment of biological processes among these genes was analysed using DAVID Functional Annotation Clustering^28^. Five Gene Ontology (GO) clusters had enrichment scores >1.3 (referring to geometrical mean of the *P* values of the pathways <0.05) in subjects with SSI (Supplementary Table S2).

The GO cluster 5 (“Lipid cluster”, *P* = 0.045, Supplementary Table S2 and Supplementary Fig. S2) included 4/5 of the top pathways that were enriched among down-regulated genes in the SR study (permuted *P* < 0.001) reported previously (Table 1)^5^. The Lipid cluster remained significant (*P* < 0.05) also after including BMI as a covariate in the explanatory model.

The common pathways in the experimental SR study and the DILGOM sample were: Cholesterol transport, Sterol transport (both *P* = 0.048), Cholesterol homeostasis and Sterol homeostasis (both with a borderline significance of *P* = 0.052) (Table 1). The genes contributing to these GO pathways in the DILGOM sample were ATP-binding cassette, sub-family G, member 1 (ABCG1), caveolin 1 (CAV1), Niemann-Pick disease, type C1 (NPC1), and Niemann-Pick disease, type C1, gene-like 1 (NPC1L1), while in the experimental SR study they were ABCA1 and NPC1. All genes and pathways of the Lipid cluster are shown in Supplementary Fig. S2.

#### Gene expression replication

Gene expression was measured in the YFS replication sample (N = 1407) from whole blood using Illumina microarrays to evaluate whether the expression of the genes found in the down-regulated pathways in DILGOM (ABCG1, CAV1, NPC1, and NPC1L1) was lower in subjects with SSI also in this sample. The ABCG1 finding replicated (*P* < 0.05) in this cohort, supporting the suppressive effect of subjective sleep loss on this cholesterol transporter.

### Serum lipids and lipoproteins

#### NMR metabolomics

Next, we examined whether the transcriptional changes were reflected in the serum lipid and lipoprotein profiles using NMR-based metabolomics analyses. This high-throughput method provides concentration information for over 200 metabolic measures, including different sized VLDL, IDL, LDL, and HDL particles, various fatty acids, amino acids, and small molecule energy metabolites^29^.

#### Concentration of lipoprotein particles and their components

**Figure 1:**
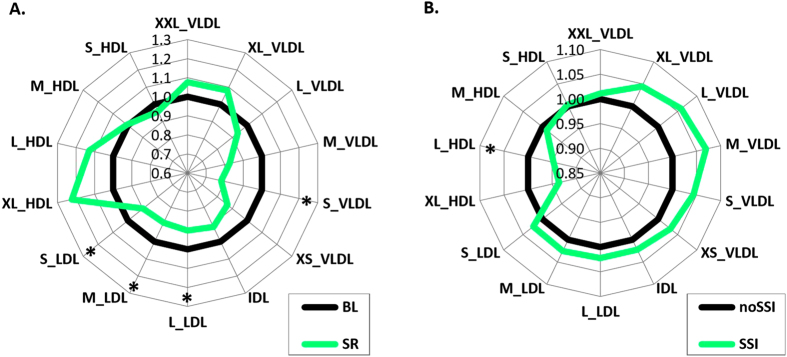
Changes in lipoprotein particles in (**A**) experimental sleep restriction (SR) and (**B**) epidemiological subjective sleep insufficiency (SSI). Concentration differences of different sized very low density (VLDL), intermediate density (IDL), low density (LDL), and high density (HDL) lipoprotein particles. (**A**) Experimental SR compared to baseline (BL, normalised to 1) (*P < 0.05, paired *t* test; N = 14). (**B**) DILGOM subjects with SSI compared to subjects without SSI (noSSI, normalised to 1) (*pointwise *P* < 0.05, linear modelling adjusting for sex and age; N = 414).

In the SR study (N = 14 cases, 6 controls), the number of small, medium, and large LDL particles (*P* after correction <0.01) as well as small VLDL particles (*P* < 0.05) was decreased after SR compared to BL, while there were no changes in the number of small and medium size HDL particles (Fig. 1A, Supplementary Table S3). Large HDL particles showed a trend of increase during SR (*P* before correction for multiple testing <0.05, not significant after correction). These changes were also reflected by changes in LDL/VLDL structural protein apoB-100. ApoB-100 levels decreased (*P* < 0.005) whereas apoA-I (major structural protein in HDL) levels did not change.

In the DILGOM sample (N = 414), the number of serum large HDL particles was lower among individuals with SSI (pointwise *P* < 0.05). SSI had an independent association to large HDL concentration also after adding BMI as a covariate in the model (*P* < 0.05). There was no significant difference in the levels of any of the LDL or VLDL subclasses, although there was a consistent trend for increase in both LDL and VLDL particles of all sizes (Fig. 1B, Supplementary Table S3).

**Figure 2:**
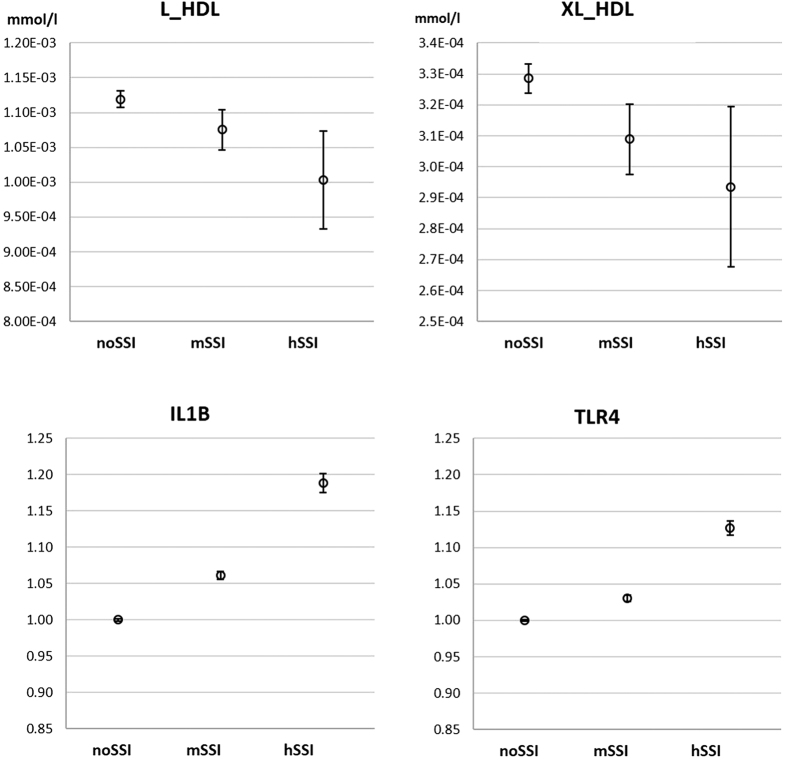
Replication in the Young Finns Study. Large (L) and extra-large (XL) HDL decreased with increasing level of subjective sleep insufficiency (SSI) (*P* < 0.005; N = 2077), whereas the expression of interleukin 1 β (IL1B) and toll-like receptor 4 (TLR4) genes was higher in subjects with SSI (*P* < 0.005 and *P* < 0.05, respectively; N = 1407). NoSSI = no or only mild SSI, mSSI = moderate SSI, hSSI = heavy SSI. HDL graphs represent mean ± s.e.m. concentrations in serum. Gene expression is shown relative to the mean expression in the noSSI group (relative mean ± s.e.m.). Effect of SSI on HDL concentrations and gene expression was modelled with linear regression adjusting for age and sex.

The lower number of large HDL particles in subjects with SSI replicated (*P* < 0.005) in the YFS sample (N = 2077) (Fig. 2). In this sample, number of XL HDL was also lower (*P* < 0.005). No differences were observed in small or medium HDLs. For the epidemiological samples, age and gender were adjusted for in the linear regression model. The decrease in large HDL was independently associated with SSI (*P* < 0.01) also when probable self-reported obstructive sleep apnoea (OSA) was included in the model.

#### Mass spectrometric measurements

In the SR study (N = 14 cases, N = 7 controls), molecular lipids were further analysed with MS-based lipidomics. Altogether 20 lipids were increased after SR in the sleep-deprived cases as compared to the controls (Supplementary Table S4). The increased lipids comprised mostly polyunsaturated phosphatidylethanolamines (PE) and phosphatidylcholines (PC) (*P* < 0.05; Supplementary Table S4).

### Network analysis on experimental SR

**Figure 3:**
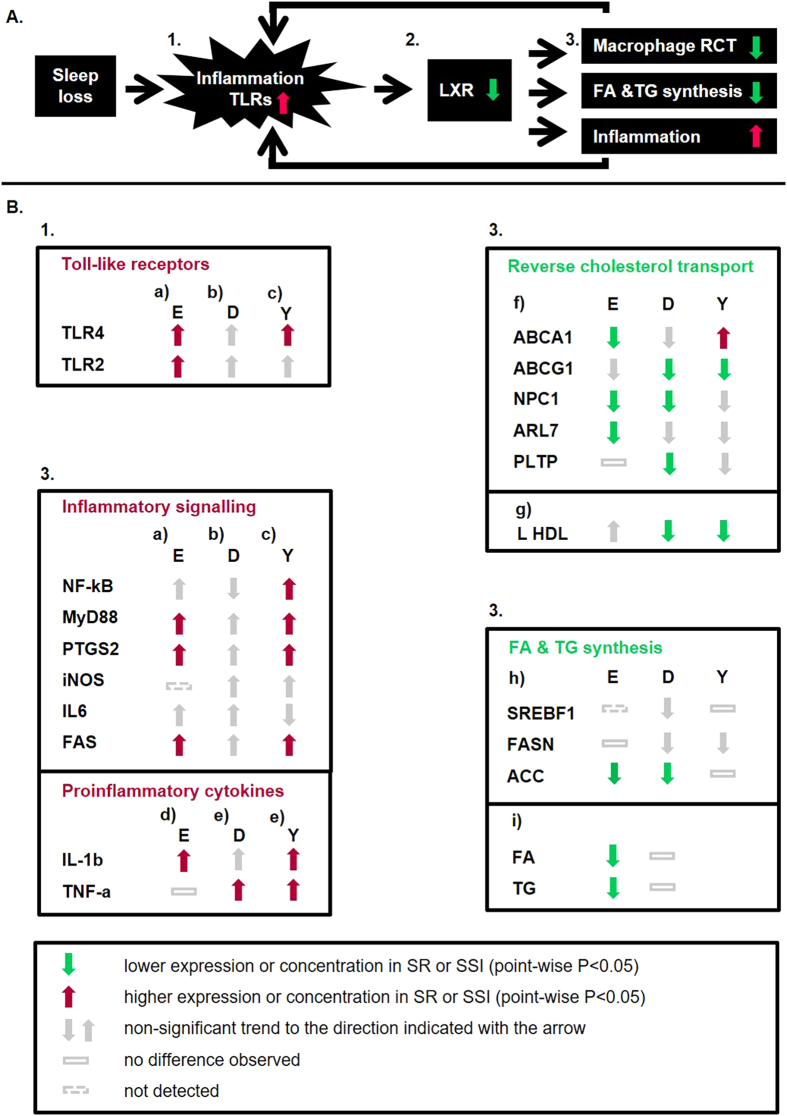
Summary of the findings from the experimental and the epidemiological studies. (**A**) Proposed model to explain the findings. **1** Sleep loss activates inflammatory responses through toll-like receptors (TLR)^5^ **2** suppressing liver X receptor (LXR) activity^33^,^34^,^35^. **3** Decreased LXR activity leads to decreased reverse cholesterol transport (RCT) and synthesis of fatty acids (FA) and triglycerides (TG), and increased immunological activation^30^,^31^,^32^. (Red arrows showing increase, green arrows decrease.) (**B**) The figure summarises our findings from transcriptomics and NMR metabolomics in experimental (E) sleep restriction (SR; N  =  21), and in the DILGOM epidemiological cohort (D; N  =  518) and Young Finns Study replication cohort (Y; N  =  2221) subjects with subjective sleep insufficiency (SSI). Numbers **1** and **3** refer to the locations in the model proposed in Fig. 3A.(a) Up-regulation of TLR and other inflammatory genes/gene pathways in SR reported in^5^. Pathway analysis for up-regulated genes in subjects with SSI in DILGOM confirmed up-regulation of B-cell activation, lymphocyte activation, and immune system development (*P*  < 0.05) also at epidemiological level. Individual genes showed only non-significant trends for higher expression in SSI. (b)TLR4 and several inflammation-related genes had higher expression among subjects with SSI in the replication sample Young Finns Study. (c) Increase in proinflammatory cytokines IL-1b and TNF-a assessed by *in vitro* stimulation of white blood cells and reported in^7^.(d) Higher expression of genes encoding for proinflammatory cytokines at epidemiological level in subjects with SSI. (e) Down-regulation of genes/gene pathways of reverse cholesterol transport assessed with transcriptomics and reported in the present publication. Concentrations of large HDL in serum measured using NMR metabolomics. (f) Acetyl-CoA carboxylase (ACC), the rate-limiting enzyme of FA synthesis, was down-regulated in SR and DILGOM SSI. No major differences were observed in the expression of other FA and TG synthesis genes in SR or SSI.(g) FA and TG measured with NMR metabolomics in experimental SR and DILGOM. Paired *t* tests used for comparing SR to BL, and linear regression used for modelling the effect of SSI on gene expression or lipid concentration, adjusting for age and sex. See abbreviations in Table 2.

The findings from the SR study suggested that cholesterol transport had declined and inflammation increased. An important transcriptional regulator of the RCT is the nuclear liver X receptor (LXR) signalling, which promotes RCT and decreases inflammation^30^,^31^,^32^. We thus hypothesized that decrease in LXR activity could be mediating the effects of sleep restriction on the immune system and metabolism (Fig. 3, Table 2). Alternatively, the activity of lipid transfer proteins could be changed. To study these hypotheses and the associations between the observed changes, we selected relevant immunological, metabolic, and sleep variables measured in the experimental SR study (listed in Supplementary Table S5), including the parameters where changes had been detected also in the epidemiological cohorts, and performed a dependency network analysis (Supplementary Fig. S3).

#### Immunological parameters

Inflammation suppresses LXR activity via toll-like receptors (TLR)^33^,^34^,^35^. We have earlier shown that the gene coding for TLR4 was up-regulated in our 5 nights’ SR protocol^5^. Also proinflammatory cytokines interleukin 1β (IL-1b) and tumour necrosis factor α (TNF-a) have been shown to suppress LXR activity^36^ (Fig. 3), and these cytokines have been consistently shown to increase in experimental sleep restriction^7^,^37^. We observed higher expression of the gene encoding for TNF-a in free-living individuals with SSI in the DILGOM sample (pointwise *P* < 0.05), and genes encoding for both IL-1b and TNF-a in the Young Finns replication sample (*P* < 0.005 and *P* < 0.05, respectively) (Figs 2 and 3). In addition to the cytokines, up-regulation of inflammation-related genes encoding for TLR4 (Fig. 2), MyD88 (an essential signal transducer in the IL-1 and TLR pathways), inducible prostaglandin endoperoxide synthase (cyclooxygenase 2, PTGS2), and Fas cell surface death receptor (FAS) – observed in the experimental SR study – replicated in the YFS sample (Fig. 3). Increased inflammation could be further augmented through decreased LXR activity^30^.

#### Dependency network analysis

In order to distinguish direct and indirect interactions of these immune, metabolic and sleep variables, we utilised undirected Gaussian graphical model where the variables are connected if and only if their partial correlation is significantly non-zero (using FDR multiple testing for the selection of edges) to visualise the dependencies as a network^38^.

The lipids that were increased after SR associated to several immunological parameters (Supplementary Fig. S3; *P* < 0.05 for all associations shown in the figure). The correlation analysis revealed significant positive associations with PE(38:3), PE(38:5e), PE(36:2e), ChoE(16:1) and B-cells. PE(38:3) was further negatively correlated with TNF-a, which was positively correlated with interleukins 18 and 1 and interferon-γ (IFNG). LXRA associated positively with B cells and TLR8, and negatively with ABCA1, NRIP1 and LXRB. TLR4 had a strong negative association with NPC1 and ABCA1 (Supplementary Fig. S3), as would be expected if LXR activity was decreased.

In the control subjects (Supplementary Fig. S4), the associations between the variables were fewer and weaker than in the experimental group. Particularly, scarce associations between the lipids and the immunological variables were observed. As the networks are baseline-corrected, they show the changes occurring between baseline and sleep restriction timepoint. As the control group was not subjected to any treatment (besides staying in the laboratory) between these timepoints, it was expected that no major changes would be detected.

### Lipid transfer protein and enzyme activities in SR

Lipid transfer proteins comprise the two key regulators of serum lipid balance between lipoproteins. However, we did not find significant changes in the activity of cholesterol ester transfer protein (CETP) or phospholipid transfer protein (PLTP) in the experimental SR study. Furthermore, no changes were observed in the activity of lecithin-cholesterol acyltransferase (LCAT), the enzyme converting HDL unesterified cholesterol to cholesteryl ester, or the atheroprotective enzyme paraoxonase 1 (PON1) linked to antioxidative properties of HDL particles.

## Discussion

The main finding of the present study was that restriction of sleep either experimentally or in natural living conditions modified lipoprotein metabolism and immune responses. The changes were detected at the level of transcriptome as well as in the circulating lipid profile. The transcriptional changes - the down-regulation of reverse cholesterol transport-related gene pathways - were similar in the relatively short term exposure of the experimental sleep restriction and in the epidemiological cohorts among subjects reporting insufficient sleep. Interestingly, the lipoprotein profile showed decreased LDL in experimental SR, resembling LDL changes in acute-phase response^39^, but in epidemiological SSI large HDL was decreased. Decrease in HDL has been regarded as one important risk factor for cardiovascular diseases^40^.

The classical view on the relationship between serum lipid levels and risk for cardiovascular events, formulated based on the findings of the Framingham study^41^ and since confirmed by many epidemiological studies, states that serum high LDL and low HDL cholesterol level is a risk combination for cardiovascular events^42^. These findings have encouraged efforts to develop treatments with the aim of either to lower LDL levels (e.g. statins) or increase HDL levels (e.g. CETP inhibitors and niacin). Particularly the latter approach has been a disappointment: pharmacological increase of serum HDL levels has not affected the risk of cardiovascular diseases^42^, indicating that the mere serum HDL cholesterol concentration is not a sufficient metrics to explain its epidemiologically verified cardioprotective effect. HDL can protect against atherosclerosis by multiple mechanisms, including the ability to efflux cholesterol from endothelial macrophages (macrophage reverse cholesterol transport)^43^, or through anti-inflammatory^44^, antioxidative^45^ and antiapoptotic^46^ pathways. A newly discovered regulation by microRNAs adds to the complexity of the task to discover mechanisms that explain the relationship between HDL and cardiovascular disease (CVD) risks^47^. Moreover, modulations in HDL particle composition can transform it to dysfunctional and, through this modulation, increase the risk of CVD^40^.

Combining the knowledge of the epidemiologically verified increased risk for CVD associated with the low HDL/high LDL lipid profile and short/insufficient sleep, we expected to measure such lipid profiles in SR and in persons with SSI. This prediction did not prove quite correct. In SR we measured, using the NMR metabolomics, decreased levels of LDL while we found no significant changes in HDL levels. In the SSI subjects we found decreased HDL levels but not significant differences in LDL levels. The one week experimental SR may have been too short to affect the number of HDL particles, while in the epidemiological samples, the number of large HDL particles was lower among those who reported insufficient sleep. Since the nature of conditions that induce insufficient sleep is often persistent^48^,^49^,^50^,^51^, we argue that the SSI subjects reporting insufficient sleep had been exposed to insufficient sleep for a longer period than those in the five day experimental sleep restriction study. We propose that the lipid profile is modulated in the course of exposure to insufficient sleep from low LDL levels at early phase of sleep insufficiency to low HDL levels upon longer exposure. However, in a cross-sectional study we cannot provide direct evidence on the duration of SSI for those who reported it. The low LDL concentration in the SR may have been induced by the inflammation, particularly by the activation of the acute phase response, induced by the sleep restriction^5^.

While some previous gene expression studies have indicated that cholesterol/lipid metabolism is regulated by sleep-wake cycle and sleep restriction could modify it^8^,^52^,^53^, detailed characterization of lipid profiles has remained scarce. It has been shown that serum triglyceride levels decreased following restriction of sleep to 4 hours on 5 consecutive nights^12^, and also that myeloperoxidase-modified LDL particles increased under similar conditions^13^. In a recent study, no changes in blood total cholesterol, LDL, HDL, or triglycerides were found after sleep restriction to 4 hours on 5 nights^14^. Two recent studies have measured lipid profiles in human blood samples using mass spectrometry analysis after polar and non-polar extraction of the samples after a short term (24 h) total sleep restriction^22^ and a partial sleep restriction for five days^23^. In both studies, the majority of the altered, identified species, consisted of lipid species (including phospholipids, sphingolipids, acylcarnitides and phosphatidylcholines). In the acute SR study^22^, only increases in lipid levels were observed after the deprivation period, while in the partial SR study^23^ also declines were recorded. In the present SR study, all significant changes from BL to SR, measured using the mass spectrometry technique, were increases, mainly in phosphatidyl ethanolamides, phophatidyl cholines, triglycerides and cholesterol esters. In spite of some discrepancies in the results, possibly explained by different extraction and other methods and/or differences in the experimental arrangements, these three independent studies point out that restriction of sleep, either acutely or partially for a longer period, significantly affects serum lipid levels, as measured using mass spectrometry. In the present study, also NMR spectroscopy measurement was able to track changes in lipid profiles under conditions of restricted sleep, further confirming that this condition modifies lipid metabolism.

The results from the gene expression pathways were more consistent than those of the lipid profiles. The cholesterol pathways that were down-regulated in SR and SSI included members of the ATP-binding cassette families A and G (ABCA1 and ABCG1). ABCA1 and ABCG1 facilitate the efflux of free cholesterol and phospholipids from macrophage-foam cells to HDL particles. Of all cholesterol carried by HDL, the proportion originating from peripheral macrophages is low compared to liver (75%) and intestine (20%) produced HDL. However, this route of removal of cholesterol (macrophage RCT) has been regarded as an important (but not only) component in retaining cholesterol balance in arterial endothelium^43^. Since ABCA1 and ABCG1 work in tandem to facilitate cholesterol removal from the macrophages^54^, the reduction in their expression would implicate a reduced potential of these monocyte-derived macrophages to egress cholesterol to HDL acceptors^55^. ABCA1 deficiency in Tangier disease leads to very low or absent HDL levels and larger VLDL particles^56^. Since the relationship between cholesterol transporter expression in white blood cells and serum HDL concentration is far from simple, it is difficult to evaluate how the observed expression changes were reflected to the flow of cholesterol from macrophages to liver and finally feces (RCT), particularly as the activities of the enzymes/transporters modulating HDL pool (CETP, PLTP, LCAT, PON1) measured in the SR study were not affected by. However, it can be noted that the observed expression changes in the cholesterol transport-related genes are compatible with those in down-regulation of LXR activity^39^,^57^ (Fig. 3).

Caveolin-1 (CAV1) is found in caveolae, cholesterol-rich membrane lipid formations that are involved in cholesterol traffic and homeostasis^58^ and regulation of inflammation^59^ among other functions. CAV1 has been proposed to participate in the regulation of cholesterol efflux to HDL^60^,^61^, possibly in interaction with ABCG1^61^. Some other studies have not found an effect^62^,^63^, and the overall effect of CAV1 on cholesterol efflux is complex^61^. The observed decrease in CAV1 expression may add to the view that reverse cholesterol transport from macrophages is compromised by insufficient sleep.

Previous studies indicate that the immune system is activated during experimental sleep restriction, and in persons who in epidemiological studies report short sleep duration (reviewed in^64^,^65^). We have earlier shown that cytokine (interleukins IL-1b, IL-6 and IL-17) release as response to *in vitro* immunological challenge is increased after sleep restriction, as well as serum acute phase protein CRP^7^. At gene expression level, the activation included increases in expression of toll-like receptors, NF-kB signalling pathway and interleukin-8 production pathways^5^.

Many acute conditions, including surgical trauma, myocardial infarction and inflammation, induce the systemic acute phase reaction (APR), with acute phase protein production in the liver as an integral part of the host defence response. In addition to changes in protein synthesis, also lipid metabolism undergoes significant modifications in APR^66^,^67^. Metabolic modifications aim at optimization of the defence, and would return to baseline level after the need for defence has disappeared^68^. However, if the inflammation-sustaining condition continues, as presumably in the epidemiological SSI groups of the present study, the metabolic modifications may develop further and become chronic^39^,^68^. Moreover, the modifications differ according to the nature of the event that aroused the reaction, e.g. surgical trauma and infection^66^,^68^. Decreases in HDL and LDL cholesterol, as well as RCT, belong to the metabolic adaptations found in APR during inflammatory attack^67^. While cytokines (including IL-1b, IL-6 and TNF-a) are central regulators of the APR, recent research suggests that an important part of the metabolic regulation in APR is channelled via nuclear receptors, including LXR, PPAR and LHR-1^39^. Thus both the down-regulation of RCT pathway and up-regulation of the inflammatory pathways could result from a decrease of LXR activity (Fig. 3).

### Methodological issues

To evaluate sleep in the epidemiological samples, we used subjective evaluation of sleep insufficiency, instead of sleep duration. While sleep duration as a measure of sleep has many advantages, in evaluation of sleep insufficiency it has one considerable disadvantage: it cannot differentiate sleep insufficiency-inducing short sleep from natural short sleep^69^.

In epidemiological studies sleep duration and self-reported sleep insufficiency have shown independent effects on cardiovascular outcomes (including hypertension, cholesterol level and cardiac events)^70^ and performance^71^. Thus, although the correlation of short sleep duration and subjective sleep insufficiency is usually high (in the present study, *P* = 2.5E-10 and β = −0.5 h in DILGOM, and *P* = 4.9E-93 and β = −0.5 h in YFS, Supplementary Fig. S5), these variables may represent partly overlapping, but separate physiological entities^69^,^70^. In epidemiological studies, a question addressing sleep need and/or subjective feeling of sleep insufficiency may help to separate natural short sleepers from those that don’t get enough sleep.

While the participants of the experimental study were carefully screened not to have any (sleep) disorders, the participants in the epidemiological studies reported different medical conditions, including sleep apnoea, which is known to be associated to both insufficient sleep and cardiovascular diseases. The evaluation of the potential effects of OSA on our results revealed that although the subjects reporting symptoms of OSA reported also more sleep insufficiency, OSA did not explain the difference in HDL, as SSI was independently associated to lower level of large HDL.

The main regulation of metabolism takes place in other tissues (e.g. liver, pancreas) than blood cells, which were used in the gene expression analyses. Thus it is unclear to which extent the results reflect changes in the actual metabolic processes. An additional source of variation lies in the differences in age and gender between the populations studied. The effects of these factors have been addressed by including them in the linear models used for statistical analyses.

Sleep loss is often accompanied with circadian misalignment, also known to cause changes in the regulation of the immune system and metabolism and associate to cardiovascular diseases^72^,^73^. In our experimental SR study, the modest delay (16 min) in circadian rhythm, measured in the morning salivary cortisol peak, is unlikely to significantly modulate the results, but the effect cannot be entirely ruled out.

In real life, people frequently experience consecutive cycles of sleep restriction and recovery sleep (e.g., shift workers, people working extended days). Often recovery sleep between two periods of sleep restriction remains incomplete and induces a carry-over effect^74^. In the SR experiment, participants were subjected to only one cycle. From this perspective, the results of the SR study may underestimate what actually happens to the cholesterol metabolism of sleep-restricted individuals in real life.

We propose that sleep restriction arouses a host defence response that shares features with APR aroused by trauma and infection. These responses modulate lipid metabolism, possibly through LXRs, and in the long run may contribute to the increased risk of cardiovascular diseases.

## Conclusion

Sleep restriction-induced decrease in expression of genes in pathways related to reverse cholesterol transport from macrophages, in combination with inflammatory activation, may at least partly explain the increased risk for cardiovascular diseases established in epidemiological studies for persons with short/insufficient sleep.

## Methods

### Samples

#### Experimental sleep restriction

An experimental schedule simulating a working week with restricted sleep was executed at the Brain and Work Research Centre of the Finnish Institute of Occupational Health (FIOH). We have earlier reported changes observed in glucose metabolism^75^, cytokines, white blood cell subpopulations, and C-reactive protein^7^, leukocyte gene expression^5^, as well as cognitive performance^76^ from this SR experiment. Study participants were men (N = 21; age 19–29 years; mean ± s.d. 23.2 ± 2.2 years; Supplementary Table S1) with regular sleep wake cycle that was checked by wrist-worn actigraphy and sleep diaries during 1–2 weeks prior to the study. In the sleep laboratory, the participants were randomly allocated to the experimental group (“cases”; N = 14) or the control group (“controls”, N = 8) (Supplementary Fig. S1). Cases spent the first two nights 8 h/night in bed (baseline, BL; from 23:00 h to 07:00 h), followed by five nights of 4 h/night in bed (sleep restriction, SR; from 03:00 h to 07:00 h). Controls spent 8 h in bed every night. One control subject was excluded after the EEG analysis as he had been sleeping less than 6 h/night during the experiment. Total sleep duration in the cases (N = 14) decreased from 7 h 22 min ± 20 min in BL to 3 h 54 min ± 5 min in SR, while it remained relatively constant, 7 h 19 min ± 16 min to 7 h 26 min ± 17 min at the same timepoints, in the controls (N = 7).

During waking, main activities of the participants included training and testing of memory and motor tasks simulating office work tasks. These tests were conducted during the day at 10:00–12:30 and 14:30–15:40, and during the sleep restriction at 00:30–01:40. In the test room the illumination ranged from 150 to 400 lux and in the living room from 350 to 600 lux. Physical exercise or leaving the sleep laboratory was not allowed during the experiment. Polygraphy was measured continuously and EEG scored according to the Rechtschaffen-Kales manual^75^,^77^. EEG data from the previous night before morning blood sample-taking for timepoints BL and SR were included in the dependency network analysis.

Prestudy mean (±s.d.) body mass index (BMI) was 23.2 (±2.4) for controls and 23.5 (±2.6) for cases. Standardised meals based on the Finnish nutrition recommendations for 18–30 years old normal-weighted men with low activity^78^ were eaten at fixed times throughout the experiment: breakfast at 08:00 h (600 kcal), lunch at 12:30 h (800 kcal), dinner at 18:30 h (700 kcal); snacks at 15:30 h (300 kcal) and 21:30 h (200 kcal). In addition, cases ate a piece of fruit (apple or orange) at 00:30 h (50 kcal) which did not exceed the estimated increase of energy expenditure during the SR^79^. No caffeine, alcohol, or tobacco was allowed during the experiment. Blood samples were collected at BL and SR timepoints at 7.30 h after fasting overnight.

Saliva cortisol was assessed 10 times per day during the experiment, and only a modest, 16 min on average, delay in the circadian phase measured by the morning peak of cortisol (from mean ± s.d. 07:39 ± 0:14 in BL to 07:55 ± 0:11 in SR) was observed in the sleep-deprived cases^75^. No changes in the overall cortisol concentration in SR compared to BL was observed^7^.

#### The DILGOM cohort

The Dietary Lifestyle and Genetic determinants of Obesity and Metabolic Syndrome (DILGOM) study was originally performed as an extension of the FINRISK 2007 study (N = 7993; age 25–74 years; from five geographical areas in Finland). The national, cross-sectional FINRISK surveys have been carried out every 5 years since 1972 to assess the risk factors of chronic diseases (e.g. CVD, diabetes, obesity, cancer) and health behaviour in the working age population in Finland. The aim of the DILGOM study (N = 5024) was to characterise risk factors for metabolic and cardiovascular diseases in the Finnish population both at the epidemiological and at the genetic level^25^. In addition to questionnaire data on health and lifestyle, a blood sample was drawn after at least 10 h of overnight fasting for genetic and biomedical analyses. Time of sampling varied from 7:11 a.m. to 11:31 a.m., with mean 8:56 a.m. (±s.d. 58 min).

NMR metabolomics and genome-wide gene expression were measured for a subsample from Helsinki metropolitan area (N = 518, age 25–74 years, 54% females, Supplementary Table S1, Supplementary Fig. S1)^80^. For the present study, these measures were correlated with a question probing subjective feeling of insufficient sleep. The question was ”Do you, in your opinion, sleep enough?”, and it had four answer options: 1) “Yes, almost always” (N = 168), 2) “Yes, often” (N = 218), 3) “Seldom or almost never” (N = 86), and 4) “I cannot say” (N = 46). Answer 4 was excluded, and answers 1–3 (N = 472) were dichotomised combining 1 and 2 to a phenotype of ‘subjective sufficient sleep’ (noSSI, N = 386) and comparing this to 3, ‘subjective sleep insufficiency’ (SSI, N = 86). Habitual sleep duration was assessed with the question “How many hours, on average, do you sleep per night?” with a free entry answer in hours.

#### The Young Finns Study (YFS) cohort

The Cardiovascular Risk in Young Finns Study (“Young Finns Study”, YFS) is a Finnish prospective multi-centre cohort^26^. It aims to study cardiovascular risk factors in children and adolescents (aged 3, 6, 9, 12, 15 and 18 at baseline). The study participants were randomly selected in 5 centres in Finland. The baseline study was conducted in 1980 (N = 3596). Sleep parameters were assessed with a questionnaire in 2007 when the study subjects were 30 to 45 years of age (Supplementary Table S1, Supplementary Fig. S1). Blood samples were collected after overnight fasting (97.4% of the subjects reported to have fasted overnight before the sample taking as requested and 1.3% not to have, while no information on fasting was obtained for 1.4%). The mean time of sampling was 9:46 a.m. (±s.d. 1:38).

In this cohort, subjective sleep insufficiency was assessed using two questions. Subjects reported their habitual sleep duration on a 10-level scale (≤5 h, 6 h, 6.5 h, 7 h, 7.5 h, 8 h, 8.5 h, 9 h, 9.5 h, or ≥10 h). They also evaluated their subjective sleep need (“How many hours of sleep do you need per day to feel well rested?”) on the same scale. A measure of subjective sleep insufficiency for each subject was formed by subtracting the sleep duration from the sleep need. Subjects (N = 2221 with SSI data) were grouped into three groups based on their level of SSI: no (or only mild) SSI (sleep need – sleep length = −1…0…1 h; N = 1825), moderate SSI (1.5–2 h; N = 304), and heavy SSI (>2 h; N = 55). Subjects sleeping more than 1 h over their subjective sleep need (N = 37) were not included in the analyses. Correlation of sleep insufficiency with sleep duration is shown in Supplementary Fig. S5).

Possible self-reported OSA was estimated using questions addressing the frequency and quality of snoring, as described earlier^81^. The questions used were “How often do you snore?”, “What does your snoring sound like?”, and “Have you noticed (or have others noticed) respiratory pauses when you sleep?”. Self-reported probable OSA was diagnosed if snoring was frequent (at least 3–5 nights per week) and either of the following was true: a) snoring was loud and irregular, with occasional respiratory pauses and/or stertorous breathing, or b) respiratory pauses at least 1–2 nights per week. The prevalence of probable self-reported OSA in the YFS sample using these criteria was 5%.

### Measures

#### Expression microarrays

##### DILGOM cohort

Genome-wide gene expression analysis was performed for 518 individuals (Supplementary Fig. S1). Gene expression was detected and data processed as described in^80^. Briefly, peripheral blood total RNA was extracted with PAXgene Blood RNA Kit (Qiagen GmbH, Hilden, Germany). RNA quantity and quality was evaluated with 2100 Bioanalyzer (Agilent Technologies, Santa Clara, CA, USA). 200 ng of total RNA from each sample was used for production of biotinylated cRNA with Ambion Illumina TotalPrep RNA Amplification Kit (Applied Biosystems, Foster City, CA, USA). 750 ng of biotinylated cRNA was hybridised onto Illumina HumanHT-12 Expression BeadChips (Illumina Inc., San Diego, CA, USA). Biotinylated cRNA preparation and hybridization onto BeadChip were done in duplicates for each sample.

Statistical analysis. All arrays were quantile normalised at the strip-level. Technical replicates were combined via a bead-count weighted average, and replicates with Pearson’s correlation coefficient <0.94 or Spearman’s rank correlation coefficient <0.60 were removed from further analysis. 472 subjects with gene expression and sleep sufficiency data were included in the final analysis. 35,420 probes were analysed.

Subjects were grouped into two groups (SSI, noSSI) based on their report of subjective sleep insufficiency. Linear regression was used to correlate RNA expression with SSI, adjusting for age and gender (R version 2.14).

### Young Finns Study cohort

Genome-wide gene expression was detected using Illumina microarrays as described in^82^. Briefly, 2.5 ml of whole blood was collected to a PAXgene Blood RNA Tubes (PreAnalytix, Hombrechtikon, Switzerland) after fasting overnight. RNA was isolated with PAXgene Blood RNA Kit (Qiagen) with DNase Set according to manufactures instructions using the QiaCube.

The concentrations and purity of the RNA samples were evaluated spectrophotometrically with NanoDrop (BioPhotomer, Eppendorf, Wesseling-Berzdorf, Germany). Samples were considered pure, if the 260/280 ratio was 1.8–2.2. The RNA isolating process was validated by analysing the integrity of RNA with the RNA 6000 Nano Chip Kit (Agilent).

The expression levels were analysed with an Illumina HumanHT-12 version 4 Expression BeadChip (Illumina Inc.) containing 47,231 expression and 770 control probes. In brief, 200 ng of RNA was reverse-transcribed into cDNA and biotin-UTP-labelled using the Illumina TotalPrep RNA Amplification Kit (Ambion); 1500 ng of cDNA was then hybridised to the Illumina HumanHT-12 v4 Expression BeadChip. The BeadChips were scanned with the Illumina iScan system.

Statistical analysis. Raw Illumina probe data was exported from GenomeStudio and analysed in R (http://www.r-project.org/) using the Bioconductor (http://www.bioconductor.org/) packages. The expression data was processed using nonparametric background correction, followed by quantile normalization with control and expression probes, using the neqc function in the limma package and log2 transformation. The expression analysis was successful for 1650 subjects.

Subjects were grouped into three groups based on their level of subjective sleep insufficiency (noSSI, moderate SSI, heavy SSI; N = 1407). Linear regression was used to correlate RNA expression with SSI, adjusting for age and gender (R version 2.14).

### Pathway analysis

The genes with differing expression levels in the SSI group compared to subjects satisfied with their sleep amount (*P* < 0.05) in the DILGOM cohort were further analysed with Database for Annotation, Visualization and Integrated Discovery (DAVID) pathway analysis (DAVID Bioinformatics Resources 6.7, NIAID/NIH, http://david.abcc.ncifcrf.gov/home.jsp). Illumina probe IDs were converted to DAVID IDs using Gene ID Conversion Tool, removing all redundancies in the original IDs. DAVID Functional Annotation Tool was used for identifying enriched biological processes among the genes with lower or higher expression in the SSI group (analysed separately; N genes 725 and 734, respectively) compared to the whole human genome^28^. Gene Ontology (GO) Biological Process annotations were used and pathways with less than 3 genes from our gene list were excluded. Other than this, default parameters were used in the analysis. Considering the hierarchical nature of GO annotations, the GO pathways were grouped using DAVID Functional Annotation Clustering. Medium classification stringency and other default parameters were used.

### NMR spectroscopy

The NMR-based analyses provide absolute quantification of multiple serum metabolites, including lipoprotein subclass distribution, fatty acids, and various small molecules including amino acids and glycolysis precursors (Supplementary Table S3). The lipoprotein subclasses were classified as follows: chylomicrons and extremely large VLDL particles (average particle diameter at least 75 nm); five different VLDL subclasses: very large VLDL (average particle diameter of 64.0 nm), large VLDL (53.5 nm), medium VLDL (44.5 nm), small VLDL (36.8 nm), and very small VLDL (31.3 nm); intermediate-density lipoprotein (IDL) (28.6 nm); three LDL subclasses: large LDL (25.5 nm), medium LDL (23.0 nm), and small LDL (18.7 nm); and four HDL subclasses: very large HDL (14.3 nm), large HDL (12.1 nm), medium HDL (10.9 nm), and small HDL (8.7 nm). The NMR-based metabolite profiling has previously been used in various epidemiological and genetic studies^83^,^84^,^85^ and details of the method have been described^25^,^29^,^86^.

Statistical analysis. The concentrations of 135 serum metabolites were assessed with NMR spectroscopy from serum samples of the experimental SR study and the epidemiological cohorts. In the SR study, SR timepoint was compared to BL with paired *t* tests. Hierarchical clustering analysis using principal components revealed that the 135 metabolites cluster into 21 clusters that explain 95% of the variance. *P* values from the *t* tests were corrected with the amount of clusters (N = 21).

In the epidemiological samples DILGOM and YFS, linear regression was used to correlate NMR metabolites with SSI, adjusting for age and gender (R version 3.0), similarly as was done in the gene expression analysis. In the YFS sample, the analyses were run also with self-reported probable OSA added in the model.

### Mass spectrometry

Lipidomic analyses were performed on serum samples from the experimental SR study at VTT Technical Research Centre of Finland. Ultra Performance Liquid Chromatography (UPLC) coupled to electrospray ionization quadrupole time-of-flight mass spectrometry (QTOFMS) was used with the established protocol^87^. Data was processed using the MZmine 2 software^88^. The data was normalised using internal standards representative of each class of lipid present in the samples. A total of 530 lipids were detected and 330 identified.

Statistical analysis. BL-normalised values of the lipids in the SR timepoint were compared between cases and controls using *t* tests.

### Lipid transfer protein and enzyme activity assays

CETP activity was analysed as the transfer/exchange of radiolabelled [14C]cholesteryl oleate between exogenously added human LDL and HDL2, as described in^89^. PLTP activity was assessed with phosphatidylcholine liposomes following an earlier reported protocol^90^. Liquid scintillation counting was used to detect radioactivity in HDL as a measure of transfer activity. LCAT activity was assessed by measuring cholesterol esterification activity using exogenous [^3^H]cholesterol-labelled HDL proteoliposome discs as the substrate^91^. Serum PON1 activity was measured using spectroscopy method and Paraoxon (diethyl-p-nitrophenyl phosphate; Sigma) as the substrate^92^.

Statistical analysis. The activities in the SR timepoint were compared to their BL using first 2-way repeated measures ANOVA with group (case vs control) and timepoint (BL vs SR) (R version 3.0), and then with paired *t* tests between the two timepoints.

### Dependency network analysis

Partial correlation network analysis using the undirected Gaussian graphical model (Genenet package^38^) was performed on baseline-normalised data for cases and controls separately, by using the log2 of the ratio between the SR and BL timepoints. Unlike the pairwise measures of associations such as Pearson correlation coefficient, partial correlation provides a stronger criterion for dependency by adjusting for confounding effects, and thus removing spurious associations to a large extent. This is particularly favourable for an integration of multiple layers of information as in our study, because it inherently filters out false positives by discovering only direct interactions with high confidence.

52 variables from the SR study were selected for the network analysis and are listed in Supplementary Table S5. The eleven lipid molecular species included in the network analysis were those lipids that showed differences (*t* test, *P* < 0.05) between cases and controls at SR timepoint for BL-normalised data. The sleep variable data used were those from the previous night before the blood sampling (second night of BL and fifth night of SR).

In these networks non-missing edges denote non-zero partial correlations (*P* < 0.05) between pairs of variables and thus imply direct interactions. The edge width is proportional to strength of dependency. The node colour corresponds to the significance and direction of regulation comparing cases versus controls. The yED graphical editor^93^ was used for network´s visualization.

### Study approval

This study was conducted according to the Declaration of Helsinki principles, and written informed consent was obtained from all participants. The experimental sleep restriction study protocol and the FINRISK 2007 study including the DILGOM subsample were approved by the coordinating ethics committee of the Hospital District of Helsinki. The Cardiovascular Risk in Young Finns Study was approved by the ethics committees of University Hospitals of Turku, Helsinki, Tampere, Kuopio, and Oulu.

## Additional Information

**Data availability**: The gene expression data has been deposited to the ArrayExpress database (accession numbers E-MEXP-3936 and E-TABM-1036 for the experimental and epidemiological data sets, respectively)

**How to cite this article**: Aho, V. *et al.* Prolonged sleep restriction induces changes in pathways involved in cholesterol metabolism and inflammatory responses. *Sci. Rep.* **6**, 24828; doi: 10.1038/srep24828 (2016).

## Supplementary Material

Supplementary Information

## Figures and Tables

**Table 1:** Lipid pathways down-regulated in experimental sleep restriction (SR) and epidemiological subjective sleep insufficiency (SSI).

Gene Ontology Pathway			Experimental SR			Epidemiological SSI	
GO ID	Name	Genes N	*P* value	Permuted *P*	Contributing genes	*P* value	Contributing genes
GO:0032365	**intracellular lipid transport**	9	1.71E-05	**0.001**	ABCA1, CPT1B	–	–
GO:0030301	**cholesterol transport**	8	1.79E-04	**0.001**	ABCA1, NPC1	**0.048**	ABCG1, CAV1, NPC1, NPC1L1
GO:0015918	**sterol transport**	8	1.79E-04	**0.001**	ABCA1, NPC1	**0.048**	ABCG1, CAV1, NPC1, NPC1L1
GO:0042632	**cholesterol homeostasis**	8	1.79E-04	**0.001**	ABCA1, NPC1	0.052	ABCG1, CAV1, NPC1, NPC1L1
GO:0055092	**sterol homeostasis**	8	1.79E-04	**0.001**	ABCA1, NPC1	0.052	ABCG1, CAV1, NPC1, NPC1L1

4/5 of the top Gene Ontology (GO) Biological Processes that were found enriched among down-regulated transcripts in the experimental SR (permuted *P* < 0.001)^5^ were also identified among the genes with lower expression in DILGOM subjects with SSI. These pathways were involved in (chole)sterol transport and homeostasis, and contributed to the “Lipid cluster” (Cluster 5, *P* = 0.045, Supplementary Fig. S2). Down-regulation of the NPC1 gene was shared in both samples.

**Table 2:** Variables in Fig. 3B.

TLR4, TLR2	toll-like receptors 4 and 2
NF-kB	nuclear factor kappa B
MyD88	myeloid differentiation primary response 88
PTGS2	prostaglandin synthase 2 = inducible cyclooxygenase
iNOS	inducible nitric oxide synthase
IL6	interleukin 6
FAS	Fas cell surface death receptor
IL-1b	interleukin 1 β
TNF-a	tumour necrosis factor α
ABCA1, ABCG1	ATP-binding cassette (ABC) transporters A1 and G1
NPC1	Niemann-Pick disease 1
ARL7	ADP-ribosylation factor-like 7
PLTP	phospholipid transfer protein
L HDL	large high density lipoprotein particles
SREBF1	sterol regulatory element binding transcription factor 1
FASN	fatty acid synthase
ACC	acetyl-CoA carboxylase
FA	fatty acids
TG	triglycerides
