# Supplementary material for: Prolonged sleep restriction induces changes in pathways involved in cholesterol metabolism and inflammatory responses
Source: Sci Rep. 2016 Apr 22;6:24828. doi: 10.1038/srep24828 (PMC4840329; doi:10.1038/srep24828)
Supplement: Supplementary Information [file srep24828-s1.pdf]

SUPPLEMENTARY INFORMATION

Prolonged sleep restriction induces changes in pathways involved in cholesterol metabolism and inflammatory responses

Authors: Vilma Aho, Hanna M. Ollila, Erkki Kronholm, Isabel Bondia-Pons, Pasi Soininen, Antti J. Kangas, Mika Hilvo, Ilkka Seppälä, Johannes Kettunen, Mervi Oikonen, Emma Raitoharju, Tuulia Hyötyläinen, Mika Kähönen, Jorma S.A. Viikari, Mikko Härmä, Mikael Sallinen, Vesa M. Olkkonen, Harri Alenius, Matti Jauhiainen, Tiina Paunio, Terho Lehtimäki, Veikko Salomaa, Matej Orešič, Olli T. Raitakari, Mika Ala-Korpela, Tarja Porkka-Heiskanen

Supplementary figures

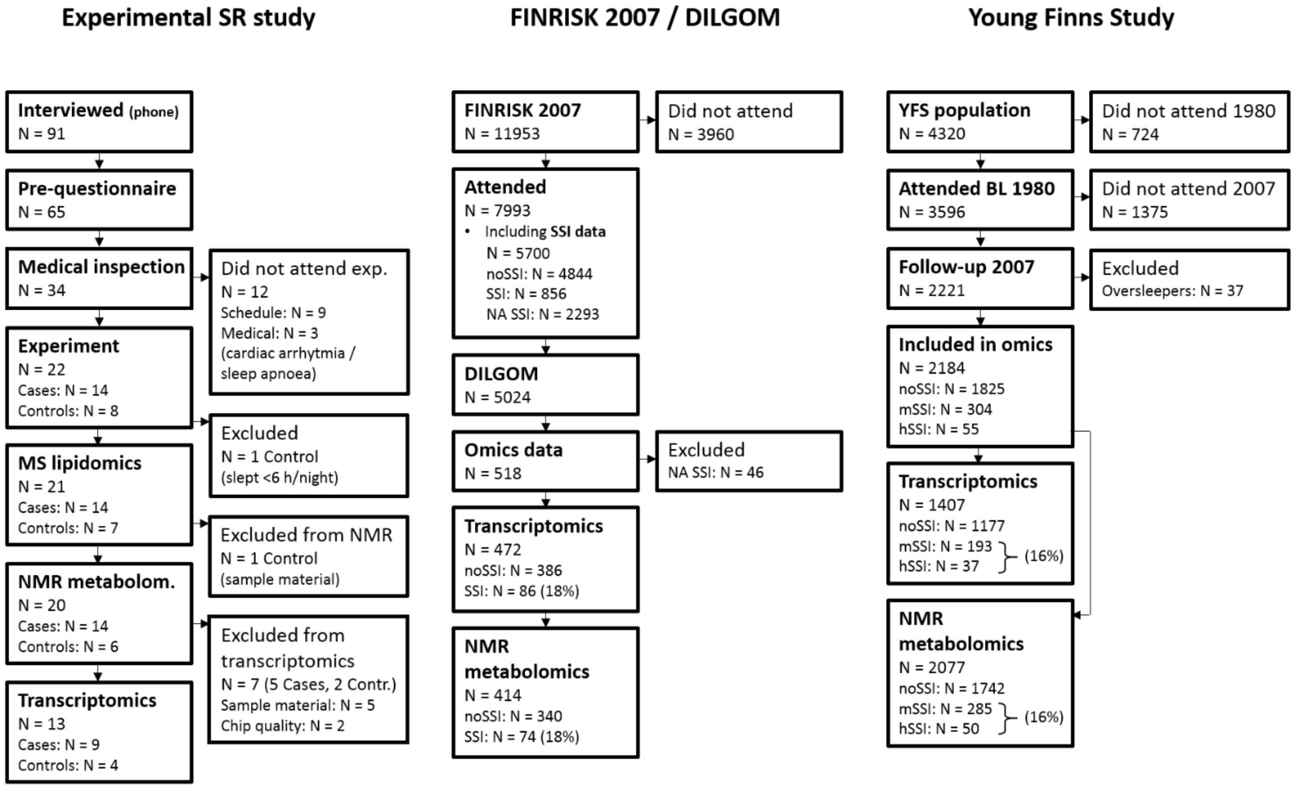

Supplementary Figure S1. Flow of the analyses.

For the experimental sleep restriction (SR) study, sleep of healthy young males was restricted to 4 h/night for 5 nights in controlled laboratory conditions and vigilance states were monitored with continuous EEG recording. In the epidemiological sample Dietary Lifestyle and Genetic determinants of Obesity and Metabolic Syndrome (DILGOM) and the replication sample Cardiovascular Risk in Young Finns Study, real-life subjective sleep insufficiency (SSI) was evaluated using questionnaires. Gene expression was analysed from peripheral blood mononuclear cells (PBMC) or whole blood using microarrays. Serum lipoprotein and lipid profiles were assessed with NMR metabolomics for all three samples and mass spectrometry (MS) for the SR study.

Pathways (N=9):	lipid homeo-stasis	chole-sterol trans-port	sterol trans-port	sterol homeo-stasis	chole-sterol homeo-stasis	lipid local-ization	chole-sterol efflux	lipid trans-port	steroid metab-olic process
GO pathway ID:	GO:0055088	GO:0030301	GO:0015918	GO:0055092	GO:0042632	GO:0010876	GO:0033344	GO:0006869	GO:0008202
GO *P* value:	8.8E-4	4.8E-2	4.8E-2	5.2E-2	5.2E-2	6.1E-2	6.5E-2	1.0E-1	2.9E-1
Genes (N=15)									
ABCG1	green	green	green	green	green	green	green	green	green
NPC1	green	green	green	green	green	green	green	green	green
CAV1	green	green	green	green	green	green	green	green	
NPC1L1	green	green	green	green	green	green		green	green
GOT1	green								
ACACA	green								
ASGR2	green								
HEXA						green			
PPARA						green		green	
LPA						green		green	
PLTP						green		green	
LEPR									green
UGT1A									green
CYP27B1									green
TSPO									green

Supplementary Figure S2. "Lipid cluster" pathways and genes.

The 9 Gene Ontology (GO) Biological Processes and 15 contributing genes in the "Lipid cluster" that was found down-regulated in DILGOM subjects with subjective sleep insufficiency (SSI). (Green = the gene is part of this GO pathway.)

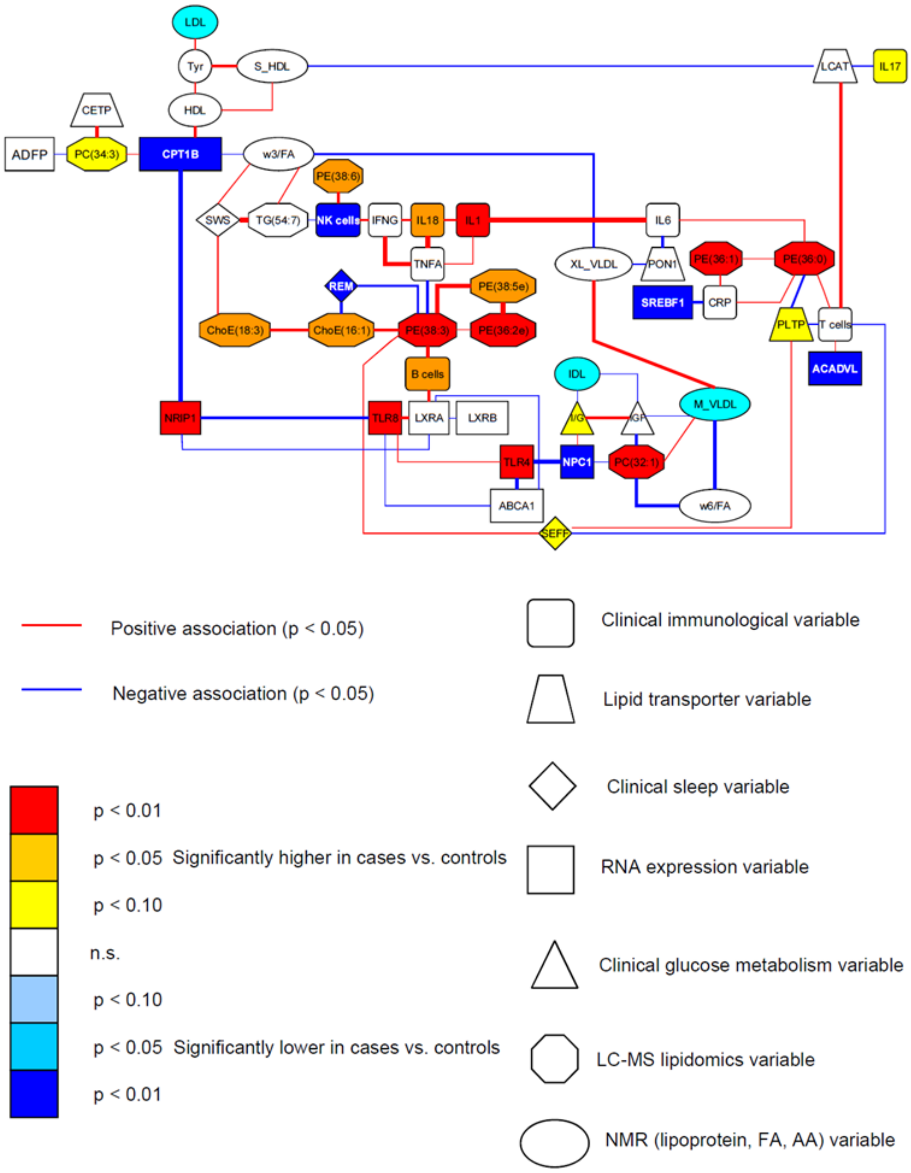

Supplementary Figure S3. Dependency network analysis using baseline-normalized variables for the experimental sleep restriction (SR).

Node shapes represent different type of variables such as clinical immunological variables, RNA expression variables, LC-MS lipidomic variables, and NMR lipoprotein variables among others (see **Supplementary Table S5**). Node colour corresponds to significance and direction of regulation comparing the experimental SR group (cases) with the control group.

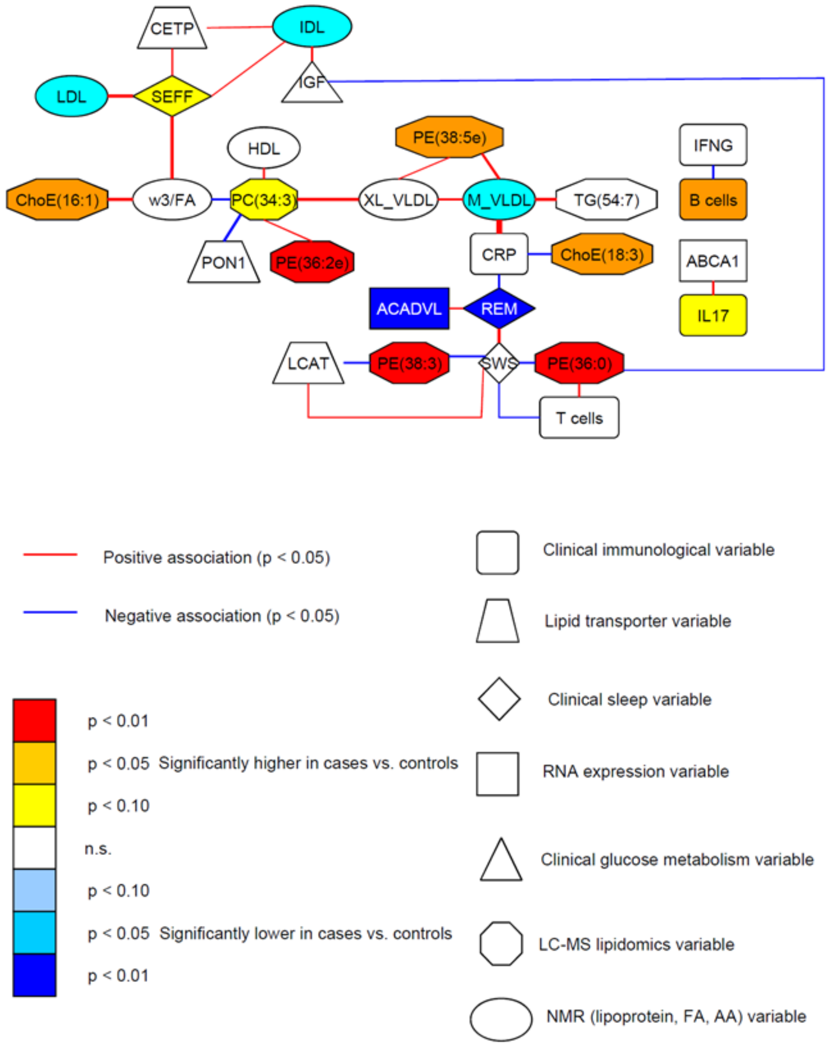

Supplementary Figure S4. Dependency network analysis of the control group in the experimental sleep restriction (SR) study.

Please see **Supplementary Figure S3** and **Supplementary Table S5** for more information.

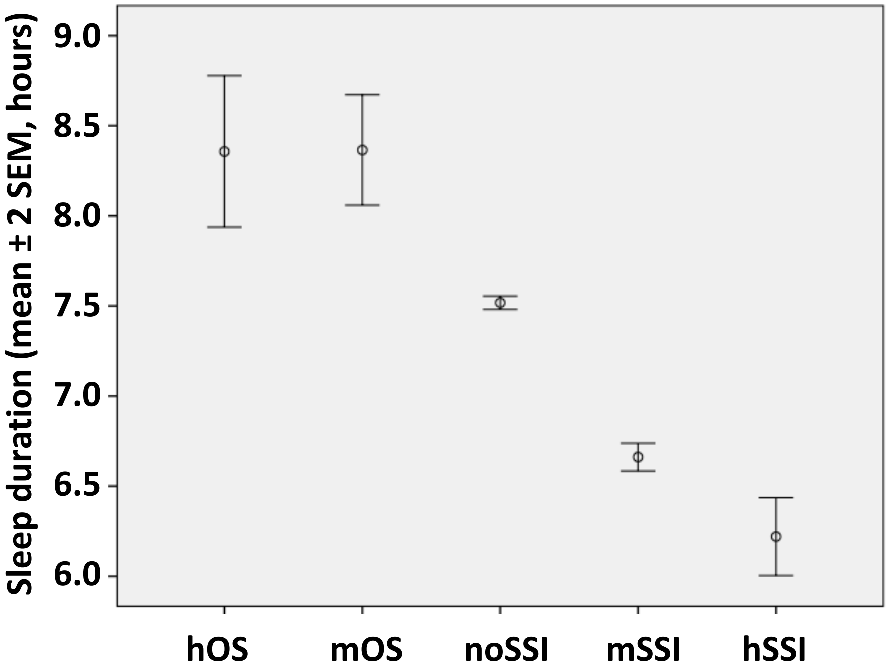

Supplementary Figure S5. Correlation of sleep insufficiency with sleep duration.

Self-reported sleep duration in subjects with different levels of subjective sleep insufficiency (SSI) or "oversleep" (OS) in the YFS sample (N=2110). Sleep duration was strongly associated to SSI (*P*=4.9E-93 and β=-0.5 h, in our linear model adjusted for sex and age, N=2077 without the OS groups). mOS = moderate oversleep (N=26), heavy oversleep (N=7), noSSI = no subjective sleep insufficiency (N=1742), mSSI = moderate SSI (N=285), hSSI = heavy SSI (N=50).

Supplementary tables

Supplementary Table S1. Characteristics of the experimental and epidemiological samples.

In the experimental sleep restriction (Exp SR) study, sleep of healthy young males (cases, N=14) was restricted to 4 h per night for 5 nights in laboratory conditions, whereas the control group (controls, N=7) had 8 hours time in bed. The epidemiological subsample of DILGOM included 18% subjects with subjective sleep insufficiency (SSI). In the Young Finns Study (YFS) replication sample, 16% had moderate (m) or heavy (h) SSI, estimated using self-reported sleep need and length.

Mean(±s.d.) values for sleep length, age, and body mass index (BMI) in each group are presented. NoSSI = subjects with no or only mild SSI. M = males, F = females.

**Exp SR (N=21)**	**N (% M/F)**	**Sleep (h:min/d)**	**Age (years)**	**BMI (kg/m^2)**
**Controls**	7 (100/0)	7:26(±0:17)	23.0(±3.0)	23.7(±2.5)
**Cases**	14 (100/0)	3:54(±0:05)	23.3(±1.7)	23.5(±2.7)
**All**	21 (100/0)	5:04(±1:43)	23.2(±2.2)	23.6(±2.3)

**DILGOM (N=472)**	**N (% M/F)**	**Sleep (h:min/d)**	**Age (years)**	**BMI (kg/m^2)**
**noSSI**	386 (48/52)	7:44(±1:13)	52.5(±13.9)	26.4(±4.9)
**SSI**	86 (37/63)	6:41(±0:51)	49.6(±12.5)	25.8(±3.9)
**All**	472 (46/54)	7:31(±1:13)	51.9(±13.8)	26.3(±4.7)

**YFS (N=2077)**	**N (% M/F)**	**Sleep (h:min/d)**	**Age (years)**	**BMI (kg/m^2)**
**noSSI**	1742 (54/46)	7:31(±0:46)	37.8(±5.0)	25.9(±4.7)
**mSSI**	285 (61/39)	6:40(±0:39)	37.0(±4.9)	26.5(±4.8)
**hSSI**	50 (58/42)	6:13(±0:46)	38.1(±5.1)	27.4(±5.3)
**All**	2077 (55/45)	7:23(±0:50)	37.7(±5.0)	26.0(±4.7)

Supplementary Table S2. Down-regulated gene clusters in epidemiological subjective sleep insufficiency (SSI).

Clusters of Gene Ontology (GO) pathways enriched among the transcripts with lower expression among DILGOM subjects with SSI (*P*<0.05). The GO Biological Processes contributing to each cluster are listed. Genes for cluster 5 ("Lipid cluster") are shown in more detail in **Fig. S2**.

**Cluster 1**		**Enrichment Score: 1.60**
**Gene Ontology annotations:**	**Genes N**	***P* Value**
angiogenesis	9	1.7E-2
blood vessel morphogenesis	11	1.9E-2
vasculature development	12	2.5E-2
blood vessel development	11	4.7E-2
**Total**	**12**	**0.025**

**Cluster 2**		**Enrichment Score: 1.52**
**Gene Ontology annotations:**	**Genes N**	***P* Value**
regulation of response to external stimulus	9	2.5E-2
regulation of response to extracellular stimulus	3	3.3E-2
regulation of response to nutrient levels	3	3.3E-2
**Total**	**9**	**0.030**

**Cluster 3**		**Enrichment Score: 1.47**
**Gene Ontology annotations:**	**Genes N**	***P* Value**
positive regulation of transcription, DNA-dependent	22	2.2E-3
positive regulation of RNA metabolic process	22	2.5E-3
positive regulation of gene expression	24	5.3E-3
positive regulation of transcription	23	7.4E-3
positive regulation of nucleobase, nucleoside, nucleotide and nucleic acid metabolic process	24	1.2E-2
positive regulation of nitrogen compound metabolic process	24	1.7E-2
positive regulation of macromolecule biosynthetic process	24	2.0E-2
positive regulation of biosynthetic process	25	2.1E-2
positive regulation of cellular biosynthetic process	24	3.2E-2
positive regulation of transcription from RNA polymerase II promoter	15	3.7E-2
positive regulation of macromolecule metabolic process	28	4.2E-2
regulation of RNA metabolic process	47	1.8E-1
regulation of transcription, DNA-dependent	45	2.3E-1
regulation of transcription from RNA polymerase II promoter	20	2.6E-1
regulation of transcription	62	3.3E-1
transcription	43	7.9E-1
**Total**	**69**	**0.034**

**Cluster 4**		**Enrichment Score: 1.45**
**Gene Ontology annotations:**	**Genes N**	***P* Value**
metal ion transport	20	7.8E-3
ion transport	28	1.2E-2

cation transport	21	2.2E-2
calcium ion transport	8	3.9E-2
di-, tri-valent inorganic cation transport	8	9.7E-2
monovalent inorganic cation transport	10	2.7E-1
**Total**	**28**	**0.035**

**Cluster 5 ("Lipid cluster")**		**Enrichment Score: 1.35**
**Gene Ontology annotations:**	**Genes N**	***P* Value**
lipid homeostasis	7	8.8E-4
cholesterol transport	4	4.8E-2
sterol transport	4	4.8E-2
sterol homeostasis	4	5.2E-2
cholesterol homeostasis	4	5.2E-2
lipid localization	8	6.1E-2
cholesterol efflux	3	6.5E-2
lipid transport	7	1.0E-1
steroid metabolic process	7	2.9E-1
**Total**	**15**	**0.045**

Supplementary Table S3. Serum metabolites in experimental sleep restriction (SR) and epidemiological subjective sleep insufficiency (SSI).

Concentrations of lipoprotein particles (VLDL, IDL, LDL, and HDL), apolipoproteins, cholesterol, fatty acids, triglycerides, phospholipids, amino acids, and small energy metabolites were measured with NMR metabolomics. Experimental SR was compared to baseline (BL) (*P* from paired *t* tests, FC = fold change). In the epidemiological sample, the concentrations were correlated to SSI adjusting for age and sex. (*P* values < 0.05 marked with blue. Green/Red = lower/higher expression among subjects after SR or with SSI.)

NMR metabolomics			Experimental SR			Epidemiological SSI		
Metabolite		Cluster	SR vs BL			SSI + Sex + Age		
Name	Short	(n=21)	*P*	*P* corrected	FC	SSI *P*	SSI β	β Unit
**Concentration of chylomicrons and extremely large VLDL particles**	**XXL_VLDL_P**	10	0.588		1.08	0.841	1.4E-09	mmol/l
Phospholipids in chylomicrons and extremely large VLDL	XXL_VLDL_PL	-	0.346		1.36	0.772	4.9E-05	mmol/l
Triglycerides in chylomicrons and extremely large VLDL	XXL_VLDL_TG	-	0.144		1.40	0.753	3.1E-04	mmol/l
Total lipids in chylomicrons and extremely large VLDL	XXL_VLDL_L	-	0.153		1.46	0.711	5.3E-04	mmol/l
**Concentration of very large VLDL particles**	**XL_VLDL_P**	-	0.748		1.08	0.569	2.5E-08	mmol/l
Phospholipids in very large VLDL	XL_VLDL_PL	-	0.367		1.29	0.684	3.0E-04	mmol/l
Triglycerides in very large VLDL	XL_VLDL_TG	10	0.495		1.15	0.599	1.5E-03	mmol/l
Total lipids in very large VLDL	XL_VLDL_L	10	0.433		1.21	0.613	2.2E-03	mmol/l
**Concentration of large VLDL particles**	**L_VLDL_P**	11	0.640		0.94	0.477	2.0E-07	mmol/l
Total cholesterol in large VLDL	L_VLDL_C	11	0.665		0.92	0.477	2.6E-03	mmol/l
Free cholesterol in large VLDL	L_VLDL_FC	12	0.901		0.98	0.527	1.2E-03	mmol/l
Phospholipids in large VLDL	L_VLDL_PL	11	0.574		0.93	0.497	2.1E-03	mmol/l
Triglycerides in large VLDL	L_VLDL_TG	11	0.714		0.95	0.468	7.7E-03	mmol/l
Cholesterol esters in large VLDL	L_VLDL_CE	11	0.379		0.86	0.424	1.4E-03	mmol/l
Total lipids in large VLDL	L_VLDL_L	11	0.730		0.95	0.479	1.2E-02	mmol/l
**Concentration of medium VLDL particles**	**M_VLDL_P**	1	0.054		0.83	0.280	8.1E-07	mmol/l
Total cholesterol in medium VLDL	M_VLDL_C	3	0.046	0.971	0.82	0.321	6.0E-03	mmol/l
Free cholesterol in medium VLDL	M_VLDL_FC	1	0.072		0.80	0.299	3.2E-03	mmol/l
Phospholipids in medium VLDL	M_VLDL_PL	1	0.026	0.536	0.81	0.265	5.3E-03	mmol/l
Triglycerides in medium VLDL	M_VLDL_TG	1	0.064		0.83	0.281	1.6E-02	mmol/l
Cholesterol esters in medium VLDL	M_VLDL_CE	3	0.031	0.645	0.84	0.350	2.8E-03	mmol/l
Total lipids in medium VLDL	M_VLDL_L	1	0.051		0.83	0.284	2.7E-02	mmol/l
**Concentration of small VLDL particles**	**S_VLDL_P**	1	5.3E-04	0.011	0.78	0.188	1.1E-06	mmol/l

Total cholesterol in small VLDL	S_VLDL_C	3	1.9E-04	0.004	0.78	0.289	5.7E-03	mmol/l
Free cholesterol in small VLDL	S_VLDL_FC	3	4.9E-04	0.010	0.75	0.210	2.8E-03	mmol/l
Phospholipids in small VLDL	S_VLDL_PL	3	3.5E-04	0.007	0.78	0.198	4.3E-03	mmol/l
Triglycerides in small VLDL	S_VLDL_TG	1	0.001	0.029	0.79	0.179	1.1E-02	mmol/l
Total lipids in small VLDL	S_VLDL_L	3	3.7E-04	0.008	0.78	0.199	2.1E-02	mmol/l
**Concentration of very small VLDL particles**	**XS_VLDL_P**	4	0.012	0.261	0.87	0.233	7.7E-07	mmol/l
Phospholipids in very small VLDL	XS_VLDL_PL	4	0.008	0.172	0.87	0.421	2.2E-03	mmol/l
Triglycerides in very small VLDL	XS_VLDL_TG	5	9.0E-04	0.019	0.80	0.141	3.9E-03	mmol/l
Total lipids in very small VLDL	XS_VLDL_L	4	0.033	0.703	0.88	0.290	9.4E-03	mmol/l
**Concentration of IDL particles**	**IDL_P**	4	0.005	0.106	0.91	0.433	1.2E-06	mmol/l
Free cholesterol in IDL	IDL_FC	4	0.018	0.372	0.92	0.562	2.2E-03	mmol/l
Phospholipids in IDL	IDL_PL	4	0.006	0.132	0.91	0.460	3.6E-03	mmol/l
Total lipids in IDL	IDL_L	4	0.008	0.175	0.92	0.484	1.3E-02	mmol/l
**Concentration of large LDL particles**	**L_LDL_P**	6	6.6E-05	0.001	0.90	0.393	2.2E-06	mmol/l
Total cholesterol in large LDL	L_LDL_C	6	4.3E-05	8.9E-04	0.90	0.420	1.4E-02	mmol/l
Free cholesterol in large LDL	L_LDL_FC	4	3.9E-04	0.008	0.92	0.419	3.5E-03	mmol/l
Phospholipids in large LDL	L_LDL_PL	6	9.1E-04	0.019	0.92	0.348	4.5E-03	mmol/l
Cholesterol esters in large LDL	L_LDL_CE	6	3.3E-05	6.9E-04	0.89	0.409	1.1E-02	mmol/l
Total lipids in large LDL	L_LDL_L	6	4.4E-05	9.2E-04	0.90	0.402	2.0E-02	mmol/l
**Concentration of medium LDL particles**	**M_LDL_P**	7	1.2E-05	2.5E-04	0.89	0.323	2.1E-06	mmol/l
Total cholesterol in medium LDL	M_LDL_C	7	5.5E-06	1.1E-04	0.88	0.319	1.1E-02	mmol/l
Phospholipids in medium LDL	M_LDL_PL	5	1.3E-04	0.003	0.92	0.275	3.1E-03	mmol/l
Cholesterol esters in medium LDL	M_LDL_CE	7	7.2E-06	1.5E-04	0.86	0.326	8.5E-03	mmol/l
Total lipids in medium LDL	M_LDL_L	7	7.3E-06	1.5E-04	0.89	0.319	1.5E-02	mmol/l
**Concentration of small LDL particles**	**S_LDL_P**	7	3.1E-04	0.006	0.90	0.375	2.2E-06	mmol/l
Total cholesterol in small LDL	S_LDL_C	7	4.5E-04	0.009	0.88	0.344	6.5E-03	mmol/l
Total lipids in small LDL	S_LDL_L	7	0.002	0.032	0.90	0.351	9.0E-03	mmol/l
**Concentration of very large HDL particles**	**XL_HDL_P**	13	0.087		1.22	0.122	-2.2E-05	mmol/l
Total cholesterol in very large HDL	XL_HDL_C	-	0.372		1.18	0.252	-8.8E-03	mmol/l
Free cholesterol in very large HDL	XL_HDL_FC	-	0.057		1.41	0.190	-2.9E-03	mmol/l
Phospholipids in very large HDL	XL_HDL_PL	13	0.015	0.311	1.28	0.086	-1.5E-02	mmol/l
Triglycerides in very large HDL	XL_HDL_TG	5	0.483		0.97	0.481	-2.4E-04	mmol/l
Cholesterol esters in very large HDL	XL_HDL_CE	-	0.422		1.14	0.242	-6.2E-03	mmol/l

Total lipids in very large HDL	XL_HDL_L	13	0.125		1.22	0.143	-2.4E-02	mmol/l
**Concentration of large HDL particles**	**L_HDL_P**	14	0.012	0.251	1.13	0.048	-5.9E-05	mmol/l
Total cholesterol in large HDL	L_HDL_C	14	1.7E-04	0.004	1.29	0.055	-2.6E-02	mmol/l
Free cholesterol in large HDL	L_HDL_FC	13	0.004	0.087	1.22	0.092	-5.4E-03	mmol/l
Phospholipids in large HDL	L_HDL_PL	14	0.078		1.09	0.052	-2.0E-02	mmol/l
Cholesterol esters in large HDL	L_HDL_CE	14	1.4E-04	0.003	1.30	0.051	-2.0E-02	mmol/l
Total lipids in large HDL	L_HDL_L	14	0.003	0.058	1.16	0.050	-4.7E-02	mmol/l
**Concentration of medium HDL particles**	**M_HDL_P**	16	0.882		1.01	0.291	-2.5E-05	mmol/l
Total cholesterol in medium HDL	M_HDL_C	16	0.206		1.07	0.172	-9.9E-03	mmol/l
Free cholesterol in medium HDL	M_HDL_FC	16	0.940		1.00	0.319	-1.5E-03	mmol/l
Phospholipids in medium HDL	M_HDL_PL	16	0.937		1.00	0.321	-5.6E-03	mmol/l
Cholesterol esters in medium HDL	M_HDL_CE	16	0.127		1.08	0.148	-8.4E-03	mmol/l
Total lipids in medium HDL	M_HDL_L	16	0.654		1.02	0.251	-1.5E-02	mmol/l
**Concentration of small HDL particles**	**S_HDL_P**	17	0.083		0.96	0.703	1.2E-05	mmol/l
Triglycerides in small HDL	S_HDL_TG	2	0.002	0.038	0.82	0.185	1.3E-03	mmol/l
Total lipids in small HDL	S_HDL_L	17	0.044	0.930	0.94	0.646	4.3E-03	mmol/l
**Serum total cholesterol**	**Serum_C**	6	0.004	0.091	0.94	0.808	1.5E-02	mmol/l
Total cholesterol in IDL	IDL_C	4	0.044	0.922	0.94	0.549	7.0E-03	mmol/l
Total cholesterol in IDL (Lipido)	IDL_C_eFR	5	6.3E-04	0.013	0.83	0.379	5.3E-03	mmol/l
Total cholesterol in LDL	LDL_C	6	1.6E-05	3.4E-04	0.89	0.373	3.1E-02	mmol/l
Total cholesterol in LDL (Lipido)	LDL_C_eFR	4	0.003	0.053	0.93	0.759	1.3E-02	mmol/l
Total cholesterol in HDL	HDL_C	14	0.161		1.04	0.096	-4.1E-02	mmol/l
Total cholesterol in HDL2 (Lipido)	HDL2_C	14	0.078		1.06	0.098	-4.2E-02	mmol/l
Total cholesterol in HDL3 (Lipido)	HDL3_C	17	0.756		0.99	0.727	1.1E-03	mmol/l
**Esterified cholesterol**	**EstC**	6	0.013	0.270	0.94	0.822	1.0E-02	mmol/l
**Free cholesterol**	**FreeC**	7	0.003	0.068	0.92	0.878	-2.7E-03	mmol/l
**Serum total triglycerides**	**Serum_TG**	1	0.011	0.234	0.85	0.319	4.1E-02	mmol/l
Triglycerides in VLDL	VLDL_TG	1	0.044	0.933	0.84	0.278	4.0E-02	mmol/l
Triglycerides in VLDL (Lipido)	VLDL_TG_eFR	1	0.013	0.272	0.81	0.237	3.7E-02	mmol/l
Triglycerides in IDL	IDL_TG	5	0.006	0.119	0.85	0.342	2.2E-03	mmol/l
**Total fatty acids**	**TotFA**	12	0.030	0.631	0.92	0.949	-1.1E-02	mmol/l
monounsaturated fatty acids; 16:1, 18:1	MUFA	12	0.003	0.070	0.86	0.914	-7.5E-03	mmol/l
omega-3 fatty acids	FAw3	18	0.433		1.04	0.973	-3.6E-04	mmol/l

ratio of omega-3 fatty acids to total fatty acids	FAw3toFA	18	0.092		1.11	0.852	1.4E-02	
omega-6 fatty acids	FAw6	3	2.6E-04	0.005	0.89	0.960	-2.5E-03	mmol/l
ratio of omega-6 fatty acids to total fatty acids	FAw6toFA	21	0.094		0.96	0.944	1.7E-02	
omega-7, omega-9 and saturated fatty acids	FAw79S	12	0.150		0.93	0.948	-8.2E-03	mmol/l
ratio of omega-7, omega-9 and saturated fatty acids to total fatty acids	FAw79StoFA	10	0.270		1.01	0.898	-3.1E-02	
18:2, linoleic acid	LA	3	2.0E-05	4.3E-04	0.87	0.877	6.3E-03	mmol/l
other polyunsaturated fatty acids than 18:2	otPUFA	3	0.470		0.98	0.941	-3.1E-03	
22:6, docosahexaenoic acid	DHA	18	0.171		0.91	0.119	-8.5E-03	mmol/l
**Mean diameter for VLDL particles**	**VLDL_D**	10	0.349		1.01	0.932	-7.9E-03	nm
**Mean diameter for LDL particles**	**LDL_D**	15	0.535		1.00	0.955	-6.6E-04	nm
**Mean diameter for HDL particles**	**HDL_D**	14	5.6E-04	0.012	1.01	0.056	-3.1E-02	nm
**Apolipoprotein A-I (Lipido)**	**ApoA1**	17	0.974		1.00	0.252	-1.8E-02	g/l
**Apolipoprotein B (Lipido)**	**ApoB**	5	1.1E-04	0.002	0.89	0.494	9.6E-03	g/l
Apolipoprotein B by apolipoprotein A-I (Lipido)	ApoBtoApoA1	5	2.8E-04	0.006	0.89	0.236	1.0E-02	
**Total phosphoglycerides**	**TotPG**	12	0.664		0.98	0.219	-1.7E-02	mmol/l
phosphatidylcholine and other cholines	PC	12	0.532		0.98	0.183	-3.7E-02	mmol/l
sphingomyelins	SM	3	0.374		0.94	0.360	3.9E-03	mmol/l
ratio of triglycerides to phosphoglycerides	TGtoPG	2	0.011	0.230	0.86	0.504	2.1E-02	
Double bond protons of mobile lipids	MobCH	2	0.002	0.046	0.91	0.667	1.8E-02	
CH2 groups of mobile lipids	MobCH2	1	0.044	0.926	0.88	0.818	1.9E-01	
CH3 groups of mobile lipids	MobCH3	1	0.020	0.427	0.92	0.972	5.5E-03	
average number of methylene groups in a fatty acid chain	CH2inFA	10	0.538		1.00	0.319	-1.3E-02	
average number of methylene groups per a double bond	CH2toDB	10	0.229		1.02	0.386	-3.5E-02	
average number of double bonds in a fatty acid chain	DBinFA	21	0.189		0.98	0.544	3.4E-03	
ratio of bisallylic groups to double bonds	BIStoDB	21	0.040	0.835	1.02	0.855	3.8E-04	
ratio of bisallylic groups to total fatty acids	BIStoFA	21	0.889		1.00	0.684	2.3E-03	
description of average fatty acid chain length, not actual carbon number	FALen	18	0.389		1.00	0.937	-1.2E-03	
3-hydroxybutyrate	bOHBut	20	0.020	0.422	0.86	0.074	-1.3E-02	mmol/l
Acetate	Ace	20	0.068		0.85	0.531	-3.3E-03	mmol/l
Acetoacetate	AcAce	2	0.083		0.74	0.267	-2.4E-03	mmol/l
Citrate	Cit	15	0.354		1.09	0.228	-1.5E-03	µmol/l
Creatinine	Crea	19	0.956		1.01	0.121	2.0E-03	mmol/l
Glucose	Glc	9	0.067		0.96	0.905	6.7E-03	mmol/l

Glycoproteins	Gp	8	0.917		1.00	0.944	-1.1E-03	mmol/l
Lactate	Lac	8	0.040	0.844	1.15	0.048	-3.8E-02	mmol/l
Pyruvate	Pyr	8	0.665		1.02	0.156	-2.2E-03	mmol/l
Urea	Urea	19	0.755		1.02	0.570	1.1E-03	mmol/l
Alanine	Ala	8	0.164		1.06	0.374	-3.5E-03	mmol/l
Glutamine	Gln	15	0.417		0.98	0.916	4.7E-04	mmol/l
Histidine	His	15	0.402		1.06	0.626	-2.6E-04	mmol/l
Isoleucine	Ile	9	0.791		0.99	0.694	3.2E-04	mmol/l
Leucine	Leu	9	0.158		1.07	0.878	-1.3E-04	mmol/l
Phenylalanine	Phe	9	0.813		1.01	0.722	-2.5E-04	mmol/l
Tyrosine	Tyr	9	0.868		0.99	0.443	-5.3E-04	mmol/l
Valine	Val	9	0.885		0.99	0.878	-3.4E-04	mmol/l

Supplementary Table S4. Changes in serum lipids after experimental sleep restriction (SR).

Baseline-normalised values for phospholipids (phosphatidyl ethanolamides, PE, and phophatidyl cholines, PC), triglycerides (TG), and cholesterol esters (ChoE) detected by mass spectrometry (MS). 10 PE, 3 PC, 2 ChoE, and one TG species increased in SR (*P*<0.05).

MS lipid					Experimental SR		
**ID**	**m/z**	**RT**	**Name**	**N**	***P* value**	**Cases mean**	**Controls mean**
1139	748.5942	375	**PE(36:0)**	52	0.0004	1.58	1.00
176	746.5722	351	**PE(36:1)**	61	0.001	1.24	0.85
78	732.5556	336	**PC(32:1)**	61	0.001	1.66	0.96
247	752.5552	381	**PE(38:5e)**	61	0.006	1.02	0.55
230	770.6054	381	**PE(38:3)**	61	0.006	1.00	0.72
932	730.5777	411	**PE(36:2e)**	42	0.008	1.02	0.65
588	640.6041	515	**ChoE(16:1)**	61	0.009	1.34	0.82
1206	704.5345	305	**unknown**	37	0.013	1.85	1.15
325	764.5381	375	**PE(38:6)**	60	0.015	1.02	0.91
1365	770.6094	358	**PE(38:3)**	41	0.021	0.97	0.74
278	664.6038	495	**ChoE(18:3)**	61	0.022	1.52	0.96
190	894.7589	474	**TG(54:7)**	61	0.024	1.23	0.81
679	846.5296	317	**unknown**	59	0.025	1.53	1.09
93	756.5545	317	**PC(34:3)**	61	0.037	1.59	1.21
1050	752.5242	279	**unknown**	29	0.040	1.67	1.21
53	768.5898	351	**PE(38:4)**	61	0.041	0.93	0.75
525	728.5599	389	**PE(36:3e)**	61	0.042	1.00	0.70
79	766.5728	345	**PC(p16:0/20:4)**	61	0.045	1.02	0.83
116	746.6041	383	**PE(36:1)**	61	0.048	0.88	0.74
581	857.7637	505	**unknown**	56	0.048	0.71	0.91

Supplementary Table S5. Variables included in the experimental sleep restriction study dependency network analysis.

Results of the analysis are depicted in **Supplementary Fig. S3 and S4**.

**Method / Data set**	**Variable**	**Abbreviation in the network**
Clinical Glucose Metab.	Insulin/glucose ratio	**Insulin/glucose**
Clinical Glucose Metab.	Insulin-like growth factor	**IGF**
Clinical Immunological	C-reactive protein	**CRP**
Clinical Immunological	T cells	**T cells**
Clinical Immunological	B cells	**B cells**
Clinical Immunological	NK cells	**NK cells**
Clinical Immunological	Interleukin 1	**IL1**
Clinical Immunological	Interleukin 6	**IL6**
Clinical Immunological	Interleukin 17	**IL17**
Clinical Immunological	Interleukin 18	**IL18**
Clinical Immunological	Tumor necrosis factor alpha	**TNFA**
Clinical Immunological	Interferon gamma	**IFNG**
EEG Sleep	Sleep efficiency	**SEFF**
EEG Sleep	REM sleep	**REM**
EEG Sleep	Slow wave sleep	**SWS**
LC-MS Lipidomics	Phosphatidylethanolamine (32:1)	**PE(32:1)**
LC-MS Lipidomics	Phosphatidylethanolamine (36:0)	**PE(36:0)**
LC-MS Lipidomics	Phosphatidylethanolamine (36:1)	**PE(36:1)**
LC-MS Lipidomics	Phosphatidylethanolamine (38:6)	**PE(38:6)**
LC-MS Lipidomics	Phosphatidylethanolamine (38:3)	**PE(38:3)**
LC-MS Lipidomics	Ether phosphatidylethanolamine (38:5e)	**PE(38:5e)**
LC-MS Lipidomics	Ether phosphatidylethanolamine (36:2e)	**PE(36:2e)**
LC-MS Lipidomics	Cholesterol ester (16:1)	**ChoE(16:1)**
LC-MS Lipidomics	Cholesterol ester (18:3)	**ChoE(18:3)**
LC-MS Lipidomics	Triglyceride (54:7)	**TG(54:7)**
Lipid Transporter	Cholesterol ester transfer protein	**CETP**
Lipid Transporter	Phospholipid transfer protein	**PLTP**
Enzyme Activity	Lecithin-cholesterol acyltransferase	**LCAT**
Enzyme Activity	Paraoxonase 1	**PON1**
RNA Expression	ATP-binding cassette transporter A1	**ABCA1**
RNA Expression	Niemann-Pick disease C1	**NPC1**
RNA Expression	Nuclear receptor-interacting protein 1	**NRIP1**
RNA Expression	Carnitine palmitoyltransferase 1B	**CPT1B**
RNA Expression	Toll-like receptor 4	**TLR4**
RNA Expression	Toll-like receptor 8	**TLR8**
RNA Expression	Acyl-CoA dehydrogenase, very long chain	**ACADVL**
RNA Expression	Adipose differentiation-related protein	**ADFP**
RNA Expression	Sterol regul. elem. bind. transcr. factor 1	**SREBF1**
RNA Expression	NR1H2	**LXRB**

RNA Expression	NR1H3	**LXRA**
NMR Lipoprotein	Extra-large Very low density lipoproteins (sum concentration incl. XXL_VLDL and XL_VLDL particle concentrations)	**XL_VLDL**
NMR Lipoprotein	Medium Very low density lipoproteins (incl. L_VLDL, M_VLDL, S_VLDL)	**M_VLDL**
NMR Lipoprotein	Intermediate density lipoproteins (incl. XS_VLDL, IDL)	**IDL**
NMR Lipoprotein	Low density lipoproteins (incl. L_LDL, M_LDL, S_LDL)	**LDL**
NMR Lipoprotein	High density lipoproteins (incl. XL_HDL, L_HDL, M_HDL)	**HDL**
NMR Lipoprotein	Small High density lipoproteins	**S_HDL**
NMR Fatty acid	Omega-3 ratio to total fatty acids	**w3/FA**
NMR Fatty acid	Omega-6 ratio to total fatty acids	**w6/FA**
NMR Amino acid	Tyrosine	**Tyr**
